# Assessment of potential jaw‐tracking advantage using control point sequences of VMAT planning

**DOI:** 10.1120/jacmp.v15i2.4625

**Published:** 2014-03-06

**Authors:** Jung‐in Kim, Jong Min Park, So‐Yeon Park, Chang Heon Choi, Hong‐Gyun Wu, Sung‐Joon Ye

**Affiliations:** ^1^ Interdisciplinary Program in Radiation Applied Life Science Seoul National University Graduate School Seoul; ^2^ Institute of Radiation Medicine Seoul National University College of Medicine Seoul; ^3^ Department of Radiation Oncology Seoul National University Hospital Seoul; ^4^ Department of Radiation Oncology Veterans Hospital Service & Medical Center Seoul; ^5^ Department of Transdisciplinary Studies and Advanced Institutes of Convergence Technology Seoul National University Suwon Korea

**Keywords:** VMAT, jaw tracking, OAR saving, dose reduction, jaw‐tracking static arc plan

## Abstract

This study aims to evaluate the potential jaw‐tracking advantage using control point sequences of volume volumetric‐modulated arc therapy (VMAT) planning. VMAT plans for patients with prostate and head and neck (H&N) cancers were converted into new static arc (SA) plans. The SA plan consisted of a series of static fields at each control point of the VMAT plan. All other machine parameters of the SA plan were perfectly identical to those of the original VMAT plan. The jaw‐tracking static arc (JTSA) plans were generated with fields that closed the jaws of each SA field into the multileaf collimators (MLCs) aperture. The dosimetric advantages of JTSA over SA were evaluated in terms of a dose‐volume histogram (DVH) of organ at risk (OAR) after renormalizing both plans to make the same target coverage. Both plans were delivered to the MatriXX‐based COMPASS system for 3D volume dose verification. The average jaw size reduction of the JTSA along the X direction was 3.1±0.9 cm for prostate patients and 6.9±1.9 cm for H&N patients. For prostate patients, the organs far from the target showed larger sparing (3.7%—8.1% on aver‐age) in JTSA than the organs adjacent to the target (1.1%—1.5%). For the H&N plans, the mean dose reductions for all organs ranged from 4.3% to 11.9%. The dose reductions were more significant in the dose regions of $D_{80},D_{90}$, and $D_{95}$ than the dose regions of $D_{5},D_{10}$, and $D_{20}$ for all patients. Likewise, the deliverability and reproducibility of jaw‐tracking plan were validated. The measured dosimetric advantage of JTSA over SA coincided with the calculated one above.

PACS numbers: 87.55.D‐, 87.55.ne

## I. INTRODUCTION

Volumetric‐modulated arc therapy (VMAT) has attracted increasing attention because of its superior delivery efficiency characterized by a shorter treatment time, lower monitor units (MU), and a dosimetric advantage over intensity‐modulated radiation therapy (IMRT).^1^, ^2^, ^3^, ^4^, ^5^, ^6^, ^7^, ^8^, ^9^ Although VMAT has much‐desired features, it is still receiving broad interest in research and development. Several investigations have been performed in order to address some known system limitations and improve planning efficiency, such as the usage of collimator rotation and interdigitizing multileaf collimator (MLC), the relevance of dose rate variation, and the impact of couch modeling on treatment accuracy.^10^, ^11^, ^12^, ^13^ The typical delivery of VMAT is based on a fixed field size and one collimator angle for each arc, depending on its projection to the target and organ at risks (OARs) during the rotation. Sometimes this fixed field size limited by MLC leaf span doesn't allow the entire target covered by one arc projection (arc field of view). The fixed field size and collimator angle for one arc does not promise sufficient flexibility for the intensity modulation. It is ideal for users to adjust those parameters, as needed, depending on the target/OAR positions, sizes, and shapes. Zhang et al.^(14)^ optimized the collimator rotation in VMAT, which provided an additional degree of freedom. They showed that this approach could improve the target coverage and normal tissue sparing compared with the standard VMAT. Recently, a jaw‐tracking method in the step‐and‐shoot IMRT was studied and demonstrated the marginal dosimetric advantage.^(15)^ Chapek et al.^(16)^ evaluated the effect of optimizing the collimator angle and of jaw tracking for three IMRT prostate plans and found the $V_{60}$ difference ranged from 3% to 9.6%. Schmidhalter et al.^(17)^ showed dosimetric benefit of jaw tracking on dynamic IMRT for prostate and head and neck cases.

In this study, a VMAT plan with fully‐tracked jaws was developed to assess a potential advantage of jaw‐tracking methods. A VMAT plan was converted into a static arc (SA) plan, which consisted of the control point sequences of VMAT as static fields. The fixed jaws in the SA plan tracked into the MLC aperture of each static field. The final plan obtained through this procedure was called the jaw‐tracking static arc (JTSA) plan. Since both SA and JTSA plans stemmed from the same optimization, the study showed only potential dosimetric advantage of jaw tracking.

## II. MATERIALS AND METHODS

### A. VMAT system

The system used was RapidArc and Eclipse 8.9 (Varian Medical Systems, Palo Alto, CA). The planning process was based on the progressive resolution optimizer (PRO2). This was the optimization algorithm used to determine the combination of field shapes and segment weights (with dose rate and gantry speed variations) which best approximate the desired dose distribution in the inverse planning problem.^(13)^ Actually, this system does not allow us to include the jaw‐tracking method during optimization. In optimization approaches, there is no restriction on collimator rotation or field size for each arc field. However, there is an autoadjust option in the TPS, which can adjust four field parameters (i.e., field size, collimator rotation, couch rotation, and isocenter position) prior to optimization. In this study, the field size was adjusted to cover the target volume and the collimator was rotated to 45°.

## B. Jaw‐tracking static arc plan (JTSA)

The VMAT plans for five prostate patients and five head and neck (H&N) patients were used for this study. The planning target volume (PTV) of H&N patients was composed of multiple treatment regions. They were characterized by a single arc with 177 control points (CPs) and adopted a field size to cover the entire target during the rotation. The VMAT plan was converted into a SA plan with 177 static fields, one at each control point. One field of SA plan corresponded to one CP in VMAT. The JTSA plan was generated with fields that closed the jaws of each SA field into the openings made by MLCs. For this purpose, we developed an in‐house program written in MATLAB (The MathWorks, Inc., Natick, MA).

**Figure 1:**
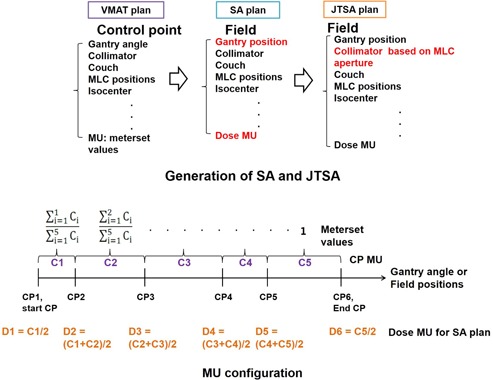
Schematic representation of SA and JTSA plans generation and MU configuration.

**Figure 2:**
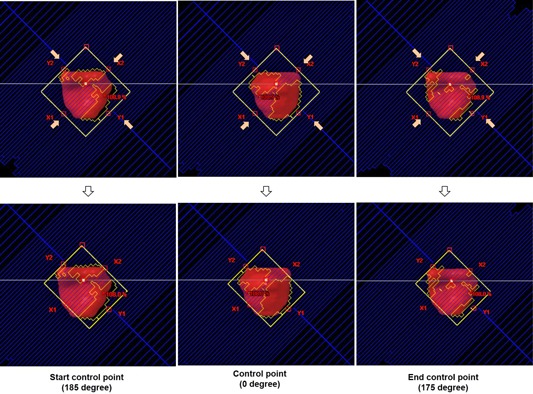
Tracking jaw for prostate patients based on MLC aperture from SA to JTSA plan.

Figure 1 depicts the schematics of SA and JTSA generation and MU configuration. In the original VMAT plan, a single arc was modeled by a sequence of CPs, defined on an aperture basis, equally spaced roughly every 2° at the end of the optimization process. Each CP MU in the DICOM plan file was a cumulative meterset value, which indicated the delivered MU up to the gantry angle. The meterset value was divided into CP MUs (C1 through C5 in Fig. 1), which was the MU to be delivered between two CPs in VMAT. Then, dose MU (D1 through D6 in Fig. 1) was calculated by using CP MUs. Finally, the dose MU was used to calculate the dose for each CP. All parameters of one CP were one static field's parameters, so that a single SA plan had 177 static fields. Likewise, both VMAT and SA plans coincided in terms of planning parameters. The jaw sizes of each static field of SA plans were tracked into the MLC aperture, as shown in Fig. 2. The X direction jaws aligned with a position of the most retracted leaf from the center. The Y direction jaws aligned with a position of the outside leaf pair. We verified that the fully‐tracked collimator to the MLC aperture boundary didn't disturb the coverage of the PTV. With this new jaw opening, a JTSA plan was generated. The JTSA plan shared the same field fluence originating from the initial VMAT optimization. The analytical anisotropic algorithm (AAA) in Eclipse (Varian Medical Systems) was used to calculate doses with a 2.5 mm gird. The MLC transmission factor in TPS was 1.7% for 6 MV. It was measured and fine‐tuned when the LINAC and TPS were commissioned.

### C. Data analysis

Since the dose distribution of the SA plan was identical to that of the VMAT plan, a comparison only between JTSA and SA was made in terms of the amount of jaw‐size reduction and dose reduction of OARs in DVH. This comparison indicated the potential advantage of jaw tracking for VMAT. Plan evaluation metrics included normal tissues doses (rectum, bladder, femoral head, and bowel for prostate patients; spinal cord, brainstem, and both parotids for H&N patients) at $D_{5},D_{10},D_{20},D_{80},D_{90},D_{95}$, and $D_{\text{mean}}$ (mean dose). The regions of $D_{5},D_{10}$, and $D_{20}$ are adjacent to PTV, while the regions of $D_{80},D_{90}$, and $D_{95}$ reached far from PTV

### D. Delivery of JTSA and SA plans

To validate the deliverability and reproducibility of the SA and JTSA, each prostate and H&N plan was delivered through a MatriXX‐based COMPASS system (IBA Dosimetry, Schwarzenbruck, Germany). The system measured the fluence of SA and JTSA plans during the delivery. The 3D dose distribution was reconstructed onto the patient anatomy based on the measured fluence and compared with planned 3D dose distribution. The comparison of dose distributions was also performed using gamma test at 2%/2 mm criteria in the COMPASS software.

## III. RESULTS

### A. Reduction in jaw sizes

**Figure 3:**
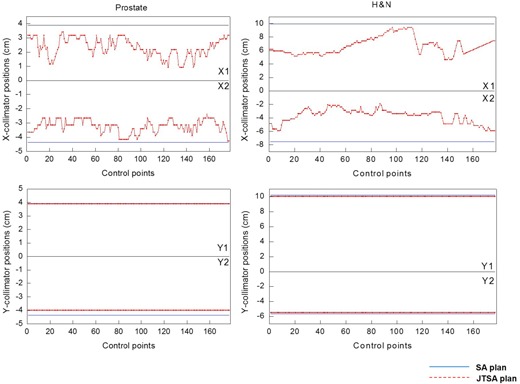
The variation of jaw size vs. 177 CPs (i.e., 177 fields) for prostate and H&N patients in both SA and JTSA plans.

The jaw sizes of each field from the SA and the JTSA plans were compared in the X and Y directions. Table 1 lists the summary of the amount of jaw size reduction. The reduction in the Y direction was minimal for both prostate and H&N cases because it was free from the modulation by MLC motion. The mean and maximum of jaw size reduction in the X direction for prostate was 3.1±0.9 cm and 5.7 cm, respectively. In the H&N cases, the mean and maximum of jaw size reduction in the X direction was 6.9±1.9 cm and 11.2 cm, respectively. The jaw size reduction was more pronounced in the multitarget H&N cases than prostate cases. Figure 3 plots the variation of jaw sizes on 177 CPs (i.e., 177 fields) of SA and JTSA plans for prostate and H&N cases, respectively. The collimator between two CPs was more variable in the multitarget H&N cases than prostate cases because the shape of target projection for H&N was more irregular through all fields and sometimes needed the isolated MLC apertures.

**Table 1:** Summary of the amount of jaw size reduction for prostate and H&N patients between SA and JSTA plans

*Patient*	*Collimator*	*X1*	*X2*	*Y1*	*Y2*	*X*	*Y*
Prostate	Max (cm)	4.5	3.8	0.4	0.3	5.7	0.5
	Min (cm)	0.0	0.1	0.0	0.0	0.2	0.1
	Mean (cm)	1.6±0.8 cm	1.6±0.8 cm	0.1±0.1 cm	0.1±0.1 cm	3.1±0.9 cm	0.3±0.2 cm
H&N	Max (cm)	9.0	5.3	0.3	0.3	11.2	0.4
	Min (cm)	0.4	0.2	0.0	0.1	1.9	0.3
	Mean (cm)	4.4±1.7 cm	2.5±1.2 cm	0.1±0.1 cm	0.2±0.1 cm	6.9±1.8 cm	0.3±0.0 cm

### A. DVH comparison

**Figure 4:**
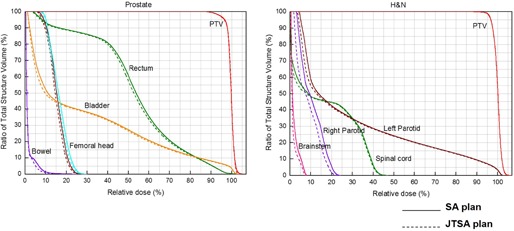
DVH comparison between SA and JTSA plans for prostate and H&N patients.

The JTSA plan was normalized to achieve the same PTV coverage as in the SA plan. The same target coverage means that the same percent of the PTV receive the prescribed dose, even though each plan had a different percentage. The normalized JTSA plan was used to evaluate the dose reduction in OARs. The normalization values were less than 1.1% and 0.7% for prostate and H&N patients, respectively, which was attributed to the decrease in collimator scatter. Even though the jaw size reduction was more significant in the H&N cases, the normalization value of H&N was lower than that of prostate since the output factor decreases rapidly in the small fields that were mostly used for prostate plans. As shown in Fig. 4, DVHs comparison proved the dose reduction in the normal structures of the JTSA plan.

Table 2 lists the summary of dose reductions and mean dose differences in various OARs averaged over five prostate and H&N patients. The structures far from the target, such as femoral heads and bowel, show more dose reduction (3.7% to 8.1%) in terms of mean dose than the structures adjacent to the target, such as the bladder and rectum (1.1% to 1.5%). The dose reduction for bladder was from 0.1% to 0.4% at $D_{5},D_{10}$, and $D_{20}$ and from 8.3% to 8.5% at $D_{80}$,

**Table 2:** Summary of dose reduction (%) and mean dose differences (%) between SA and JTSA plans in various OARs averaged over five prostate and H&N patients

*Patient*	*Structures*	*Volume*	$D_{5}$	$D_{10}$	$D_{20}$	$D_{80}$	$D_{90}$	$D_{95}$	$D_{mean}$
Prostate	Bladder	181.5	−0.1±0.2	−0.2±0.5	−0.4±0.8	−8.3±5.6	−8.4±5.7	−8.5±7.1	−1.5±0.8
		p ‐ value	0.50	0.39	0.28	<0.05	<0.05	0.05	<0.05
	Rectum	115.2	−0.1±0.1	−0.2±0.1	−0.5±0.4	−1.9±0.5	−5.0±1.6	−9.5±4.3	−1.1±0.3
		p ‐ value	0.27	<0.05	0.05	<0.05	<0.05	<0.05	<0.05
	Right Femoral Head	64.2	−3.1±1.2	−3.2±1.2	−3.5±1.3	−4.5±1.6	−5.2±2.0	−5.6±2.5	−3.8±1.3
		p ‐ value	<0.05	<0.05	<0.05	<0.05	<0.05	<0.05	<0.05
	Left Femoral Head	62.8	−3.0±0.9	−3.1±0.8	−3.4±0.9	−4.3±1.1	−4.6±1.0	−4.8±1.0	−3.7±0.9
		p ‐ value	<0.05	<0.05	<0.05	<0.05	<0.05	<0.05	<0.05
	Bowel	283.0	−9.3±8.2	−8.7±2.8	−10.9±4.3	−4.7±1.9	−4.5±1.7	−4.5±1.5	−8.1±2.4
		p ‐ value	<0.05	<0.05	<0.05	0.05	<0.05	<0.05	<0.05
H&N	Left Parotid	20.2	−2.6±3.2	−2.9±3.1	−3.6±3.2	−11.0±6.4	−11.8±6.5	−12.8±6.6	−4.9±3.8
		p ‐ value	0.14	<0.05	<0.05	<0.05	<0.05	<0.05	<0.05
	Right Parotid	20.7	−2.0±3.4	−2.6±3.9	−3.2±4.7	−10.7±16.9	−11.3±16.3	−12.2±16.2	−5.0±7.3
		p ‐ value	0.14	0.08	<0.05	<0.05	<0.05	<0.05	<0.05
	Spinal cord	24.9	−1.7±2.4	−2.4±3.1	−2.8±3.6	−17.4±3.3	−21.4±11.5	−25.3±16.2	−4.3±3.5
		p ‐ value	0.10	0.10	0.09	<0.05	<0.05	<0.05	<0.05
	Brainstem	29.0	−4.3±3.1	−7.5±7.7	−12.1±13.5	−21.5±8.9	−21.8±9.5	−21.5±8.9	−11.9±5.3
		p ‐ value	<0.05	<0.05	<0.05	<0.05	<0.05	<0.05	<0.05

$D_{90}$, and $D_{95}$, respectively. There was no significant difference at $D_{5},D_{10}$, and $D_{20}$, (p>0.05), which meant almost no influence of jaw tracking on $D_{5},D_{10}$, and $D_{20}$ for the bladder. The dose reduction for the rectum was from 0.1% to 0.5% at $D_{5},D_{10}$, and $D_{20}$ and from 1.9% to 9.5% at $D_{80},D_{90}$, and $D_{95}$, respectively. There was no significant difference for $D_{5}\;(p>0.05)$. The dose reduction for both femoral heads was from 3.0% to 3.5% at $D_{5},D_{10}$, and $D_{20}$ and from 4.3% to 5.6% at $D_{80},D_{90}$, and $D_{95}$, respectively. The dose reduction for the bowel was from 8.7% to 10.9% at $D_{5},D_{10}$, and $D_{20}$ and from 4.5% to 4.7% at $D_{80},D_{90}$, and $D_{95}$, respectively. Dose reduction in JTSA plans was statistically significant in the most of comparisons (p<0.05).

In the multitarget H&N cases, the mean dose reduction in JTSA was from 4.3% to 11.9% for all structures (see Table 2). The reduction at $D_{80},D_{90}$, and $D_{95}$ was significant for all structures compared to that at $D_{5},D_{10}$, and $D_{20}$. Dose reduction in JTSA plans was statistically significant at $D_{80},D_{90}$, and $D_{95}\;(p<0.05)$. The dose reduction for both parotids was from 2.0% to 3.6% at $D_{5},D_{10}$, and $D_{20}$ and from 10.7% to 12.8% at $D_{80},D_{90}$, and $D_{95}$, respectively. At $D_{5}$ of the left parotid and at $D_{5}$ and $D_{10}$ for the right parotid, there was no significant difference (p>0.05). The dose reduction for the spinal cord was from 1.7% to 2.8% at $D_{5},D_{10}$, and $D_{20}$ and from 17.4% to 25.3% at $D_{80},D_{90}$, and $D_{95}$, respectively. At $D_{5},D_{10}$, and $D_{20}$, there was no significant difference (p>0.05). The dose reduction for the brainstem was from 4.3% to 12.1% at $D_{5},D_{10}$, and $D_{20}$ and from 21.5% to 21.8% at $D_{80},D_{90}$, and $D_{95}$, respectively. Dose reduction in JTSA plans was statistically significant in the most of comparisons (p<0.05).

### C. 3D dose comparison

The 3D doses based on measured fluences for both SA and JTSA plans showed good agreement with calculated doses (i.e., plans), which was over 95% gamma passing rate with 2%/2 mm criteria in COMPASS analysis, as shown in Table 3. The dose reduction estimated from the measurements coincided with the calculated values, as shown in Table 4. For the prostate patient, the difference between the measurement and calculation was less than 1.5%, except for the bowel structure. The bowel structure was located far away from the target, which had a very low dose (less than 5% of prescription dose). For the H&N patient, the difference between the measurement and calculation was less than 1.4%.

**Table 3:** Summary of gamma passing rate with 2%/2 mm between plan and measurement for both plans (SA and JTSA) in various OARs of prostate and H&N patients

*Prostate*	*Body*	*Bladder*	*Rectum*	*Right Femoral Head*	*Left Femoral Head*	*Bowel*
SA	99.4%	96.0%	95.5%	100.0%	100.0%	100.0%
JTSA	99.4%	95.7%	96.0%	100.0%	100.0%	100.0%
*H&N*	*Body*	*Left Parotid*	*Right Parotid*	*Spinal cord*	*Brainstem*	–
SA	99.3%	96.8%	97.3%	98.5%	100%	–
TSA	99.2%	97.2%	95.6%	99.9%	100%	–

**Table 4:** Summary of dose reduction (%) and mean dose differences (%) between SA and JTSA plans from COMPASS measurement and TPS calculation in various OARs one prostate and one H&N patient

*Patient*	*Structures*	*Comparison*	$D_{5}$	$D_{10}$	$D_{20}$	$D_{80}$	$D_{90}$	$D_{95}$	$D_{mean}$
Prostate	Bladder	Measurement	−0.5	−1.2	−1.5	−14.6	−9.4	−8.5	−2.4
		Calculation	−0.3	−1.2	−1.9	−14.9	−10.1	−6.6	−2.7
	Rectum	Measurement	−0.6	−0.8	−1.7	−2.2	−6.3	−11.5	−1.8
		Calculation	−0.2	−0.3	−1.2	−2.1	−5.6	−9.0	−1.6
	Right Femoral Head	Measurement	−2.3	−3.2	−3.7	−6.0	−7.3	−8.9	−4.5
		Calculation	−3.5	−3.6	−4.3	−5.9	−7.5	−8.6	−4.8
	Left Femoral Head	Measurement	−3.1	−3.0	−3.8	−5.6	−6.1	−6.7	−4.4
		Calculation	−2.9	−3.3	−4.0	−5.9	−6.2	−6.4	−4.5
	Bowel	Measurement	−18.1	−10.1	−8.5	3.7	1.2	0.9	−6.8
		Calculation	−23.1	−12.3	−7.2	−4.4	−4.4	−4.9	−11.8
H&N Left	Left Parotid	Measurement	−5.7	−4.6	−4.4	−7.8	−8.5	−9.3	−5.3
		Calculation	−5.7	−4.8	−4.6	−7.2	−8.1	8.8	−5.5
	Right Parotid	Measurement	−0.3	−0.3	−0.7	−2.1	−1.5	−3.5	−1.5
		Calculation	−0.1	−0.5	−0.8	−1.6	−2.3	−2.9	−1.2
	Spinal Cord	Measurement	−6.3	−7.1	−8.5	−16.4	−38.5	−50.1	−10.2
		Calculation	−5.8	−7.3	−8.7	−18.0	−40.2	−51.0	−9.8
	Brainstem	Measurement	−5.4	−6.2	−6.3	−19.2	−22.1	−23.8	−10.3
		Calculation	−5.4	−6.3	−6.8	−20.4	−23.1	−25.6	−9.7

## IV. DISCUSSION & CONCLUSION

In this study, the gantry rotation speed and the maximum jaw speed varied from 0.5° to 4.8°/sec and 1.0 cm/sec, respectively. During VMAT delivery, the machine controller moved the gantry at the maximum speed as much as possible to minimize the treatment time. In this circumstance, the achievable maximum jaw change was 0.4 cm at the maximum gantry speed between two control points of 2°. TrueBeam (Varian Medical Systems), which allows jaw tracking, has the maximum gantry speed of 6°/sec and the maximum jaw speed of 2.5 cm/sec. Thus, the achievable maximum jaw change is 0.8 cm at the maximum gantry speed between two control points. In our JTSA plan, one side of the jaw size change between fields was from 0 cm to 5.2 cm and from 0 cm to 8.1 cm for prostate and H&N patients, respectively. This range of the jaw size change was over the achievable value under the current machine constraints. Thus, this fully jaw tracking could be the potential dosimetric advantage in OAR over the current jaw‐tracking method of TrueBeam. It gained more benefits in the dispersed target (multitargeted H&N) than the globular shape's target, such as the prostate. To take advantage of jaw tracking, it is necessary to consider mechanical constraints of radiation therapy machine in jaw‐tracking optimization.

We investigated only the 6 MV beam having a MLC transmission factor of 1.7% that was comparable to others.^(17)^ The main component influencing the dose reduction is the MLC transmission factor. A high‐energy photon beam has a higher transmission factor. Thus a JTSA plan for a high‐energy photon beam should be more advantageous in reducing OAR doses than for a low photon energy beam.

We developed a method to generate a jaw‐tracking VMAT plan from a current jaw fixed VMAT plan. Its deliverability and reproducibility were validated by reconstructing 3D dose distributions in CT images. The measured amount of dose reduction achieved by the jaw‐tracking method was almost the same as one calculated by plan comparison.

## ACKNOWLEDGMENTS

This work was supported by the National Research Foundation of Korea (NRF) grants (No. 800‐20130103 and 490‐20130047) and the Korea Radiation Safety Foundation grant (490‐20130038) funded by the Korea government. We would like to thank the anonymous referees and the Associate Editor for their valuable suggestions, which have improved this paper.

## Supporting information

Supplementary Material

Supplementary Material

Supplementary Material

Supplementary Material
