# Supplementary figures and images for: Assessment of potential jaw‐tracking advantage using control point sequences of VMAT planning
Source: J Appl Clin Med Phys. 2014 Mar 6;15(2):160–8. doi: 10.1120/jacmp.v15i2.4625 (PMC5875480; doi:10.1120/jacmp.v15i2.4625)

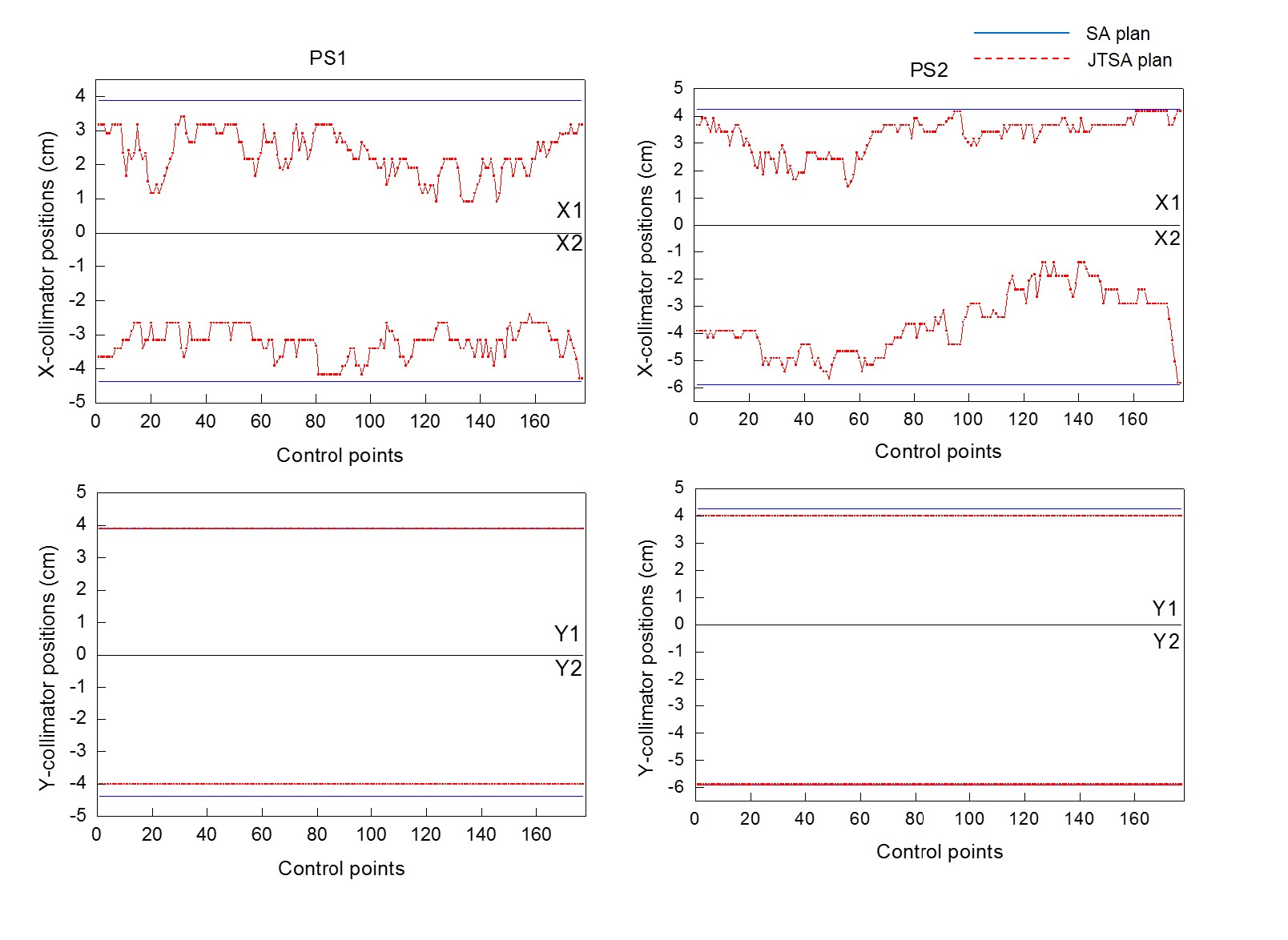

Supplement: Supplementary file 1 — Supplementary Material [file ACM2-15-160-s001.jpg]

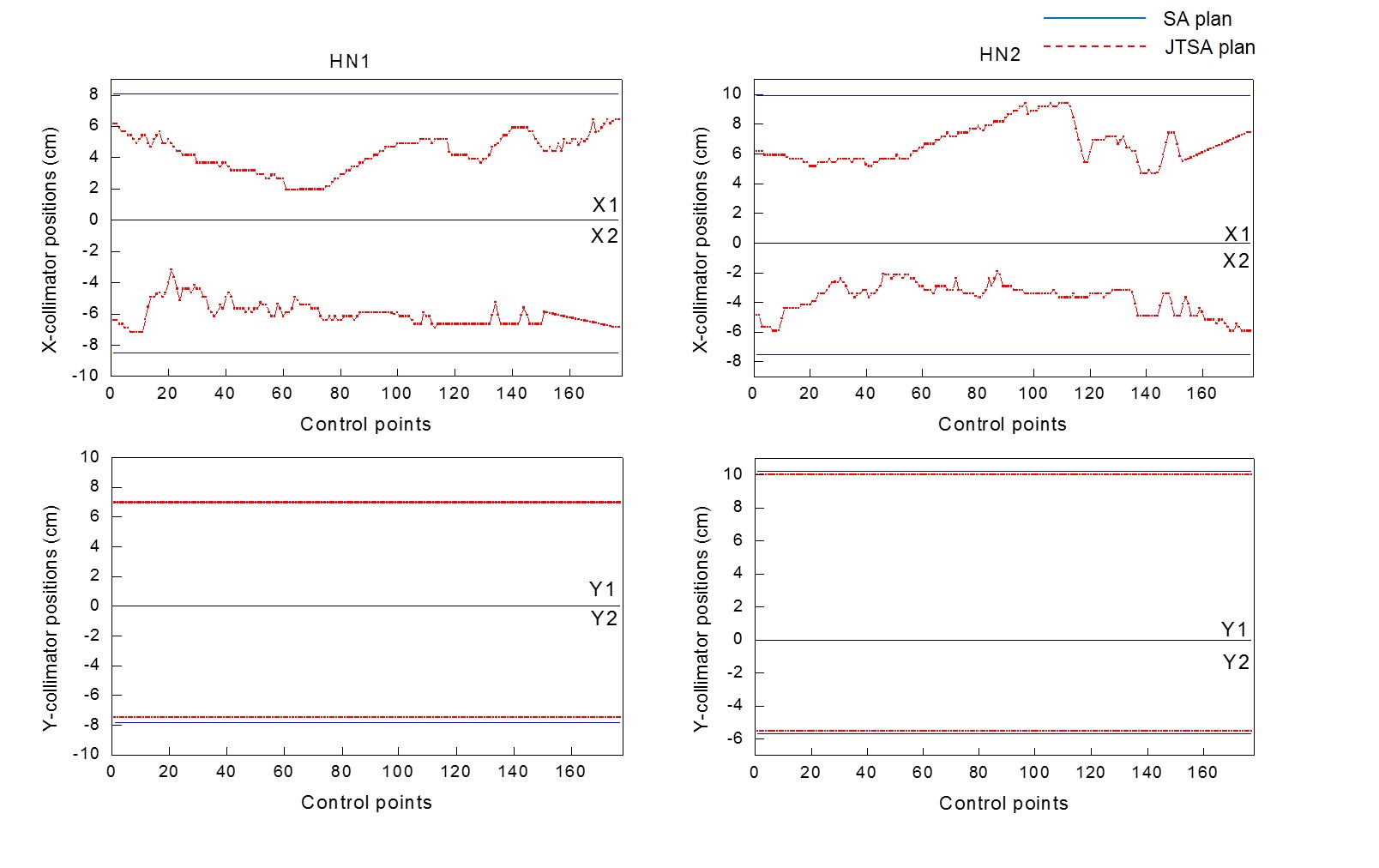

Supplement: Supplementary file 2 — Supplementary Material [file ACM2-15-160-s002.jpg]

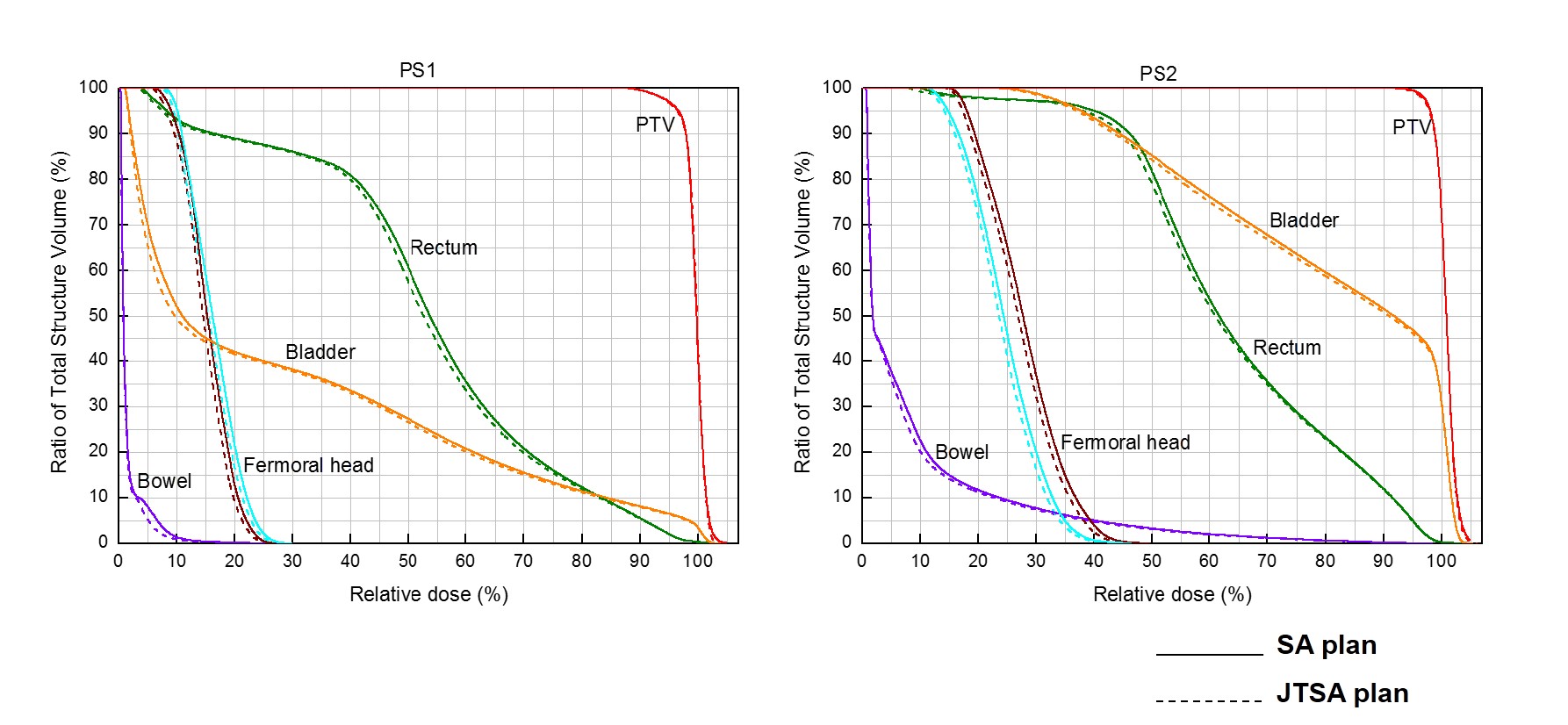

Supplement: Supplementary file 3 — Supplementary Material [file ACM2-15-160-s003.jpg]

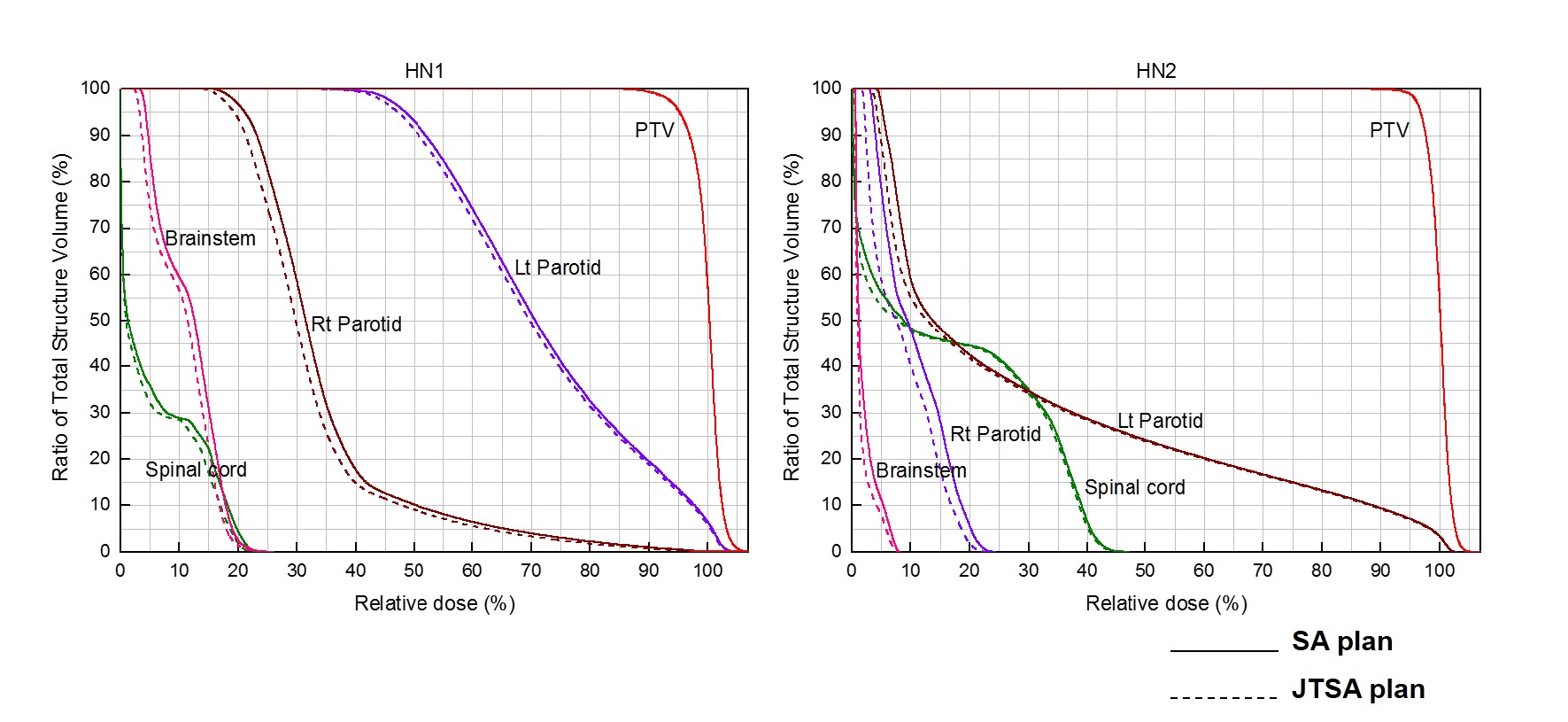

Supplement: Supplementary file 4 — Supplementary Material [file ACM2-15-160-s004.jpg]
